# The case manager through the eyes of the users: benefits and failures of a French case management experimentation

**Published:** 2012-09-04

**Authors:** Frederic Balard, Dominique Somme

**Affiliations:** Anthropologist, Fondation Nationale de Gérontologie, Paris; Hôpital Européen Georges Pompidou, Paris

**Keywords:** case management, user’s representations, qualitative analysis

## Abstract

**Purpose:**

The PRISMA France project consists in the implementation of a global services integration in which a case management services is dedicated to old people with complex needs. One of the purposes of the research team was to analyze the representations of the users, i.e., the old people and their proxies, of the case management service through their own experience of profane expert.

**Theory:**

According to the literature that deals with qualitative interviewing, we consider that the users of case management are able to express themselves on the benefit and the failures of the case management services even if some of them suffer from cognitive impairments.

**Methods:**

In this view, 30 comprehensive and semi-structured talks were done, 19 old people and 11 proxies. All the interviews were face to face interview and were done by an anthropologist. The interviews were transcribed verbatim and analyzed by focusing on the node of sense in the user’s words.

**Results and conclusions:**

On one side, the proxies describe the case manager as the one who give answers face to the complexity of the system. On the other side, the old people insisted on the relationship established with their case manager. She/he is the one who listens and asks questions in order to help them.

**Discussion:**

The analysis of the user’s representations raises three main points:

- Is the main role of the case manager is to bring an answer to the failures of the system?
- By basing their representation of the case management on relational continuity, the old people reduce the work of the case manager of the work he/she done at home. So, when the presence of the case manager diminished, old people think that his/her work in not efficient.
- How the policy makers could include the user’s points of view in the implementation of such kind of service?

Powerpoint presentation available at http://www.integratedcare.org at congresses – San Marino – programme.

